# Male genital lichen sclerosus and associated symptoms range and severity: Results of a questionnaire study

**DOI:** 10.1002/ski2.246

**Published:** 2023-05-12

**Authors:** Manu Shah, Birgitta van Bodegraven

**Affiliations:** ^1^ Department of Dermatology Burnley General Teaching Hospital Burnley UK; ^2^ British Association of Dermatologists London UK

## Abstract

**Background:**

Male genital lichen sclerosus (MGLS) is a chronic inflammatory condition most often seen in uncircumcised men. It has an association with squamous cell carcinoma of the penis and causes significant morbidity in many men. It may present with a range of symptoms, notably male dyspareunia. The full range of symptoms in individuals has yet to be elucidated.

**Aim:**

To identify the range and severity of patient's symptoms using a questionnaire to quantify symptoms, including sexual function and urinary symptoms. Patients with MGLS were compared against patients diagnosed with other genital skin conditions (non‐MGLS).

**Methods:**

Retrospective review of patients attending a specialist male genital skin clinic. A questionnaire, where symptoms were ranked between 0 (not present/no effect) and 10 (severe effect) was presented as part of the clinical assessment. Clinical diagnosis of MGLS or non‐MGLS was made at the assessment.

**Results:**

Sixty four patients attending the clinic completed the questionnaire, and 50 patients were diagnosed with MGLS. Splitting of skin (61.0%), soreness (61.0%), and unusual appearance or colour of penis (57.8%) were the most frequently reported physical symptoms in patients with MGLS. Non‐MGLS patients reported these same symptoms in 35.7%, 35.7%, and 50.0% of cases respectively.

**Conclusion:**

Administering a simple numeric questionnaire for patients with MGLS has revealed multiple symptoms experienced by most patients. Scoring these symptoms allows the clinician to focus on the problems that most concern the individual patient, rather than just focussing on the physician's assessment of clinical disease.

## Graphical abstract

Male genital lichen sclerosus (MGLS) is a complex chronic inflammatory condition. The range and severity of patient's genital symptoms was assessed using a structured questionnaire. MGLS patients suffer from multiple symptoms, including soreness and splitting of skin. Use of the questionnaire and greater understanding of associated symptoms enables clinicians to focus on patient's concern as well as the level of clinical disease.

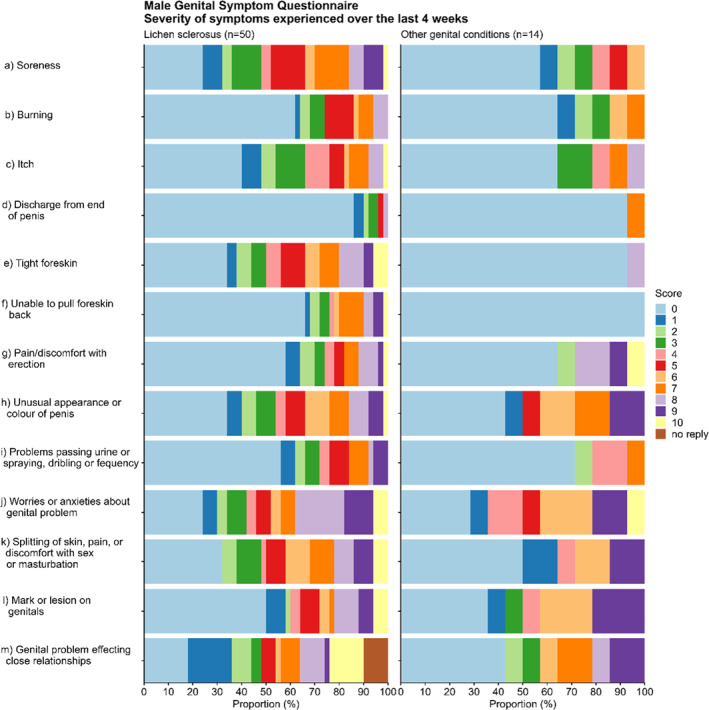

**What is already known about this topic?**

- Male genital lichen sclerosus (MGLS) is a complex, chronic inflammatory disorder that causes significant morbidity, especially dyspareunia.
- A range of symptoms have been described but not previously categorized.

**What does this study add?**

- A linear self‐reported symptom scale in MGLS reveals patients suffer multiple symptoms with a median of seven reported symptoms. Soreness is the most common symptom.
- MGLS is associated with an abnormal shaped urinary meatus.
- Understanding which symptoms are associated with MGLS enables clinicians to focus on the patient's concerns as well as the level of clinical disease.
- A structured questionnaire delivered before treatment and on follow up could be helpful in determining the patient's priorities, assessing progress and in managing symptoms.

## 1 INTRODUCTION

Male genital lichen sclerosus (MGLS) is a chronic inflammatory condition that may present with a range of symptoms related to the inflammatory process, scarring or urological issues. There is an association with subsequent incidence of squamous cell carcinoma of the penis. In recent years male dyspareunia has been highlighted as being the most significant symptom men with lichen sclerosus experience.^1^, ^2^, ^3^ The exact cause of the condition is not fully understood but there is increasing evidence for chronic exposure to urine under occlusion due to urinary micro‐incontinence.^4^, ^5^, ^6^ Symptoms may be ‘an expression of preputial and urethral dysfunction’.^2^

Although previous studies have mentioned the types of symptoms experienced by patients with MGLS, they have not focused on the severity of symptoms or whether patients experience multiple symptoms simultaneously. The importance of individual symptoms in patients has not been previously studied and insights here can provide clinical and diagnostic context which will benefit the patient. This study aimed to evaluate the range and severity of symptoms reported by patients with MGLS and compare those to patients presenting with similar symptoms but without a clinical MGLS diagnosis.

## 2 PATIENTS AND METHODS

Patients attending an English regional specialist male genital skin clinic presenting with genital symptoms were studied. All patients were new patients seen prospectively between July 2018 and February 2022. Enquiries about genital symptoms were made at presentation which had been standard clinical practice in the clinic for several years. Patients were asked to score all symptoms they had experienced during the previous 4 weeks. Diagnosis was based on the clinical experience of the lead clinician (MS). The questionnaire was designed to cover symptoms previously highlighted in published studies and supplemented with clinical knowledge. Patients were asked to score 12 symptoms between 0 (no effect) and 10 (severe effect) and were able to add and score additional symptoms not listed.

The questionnaire was divided into three sections. The first section concerned any genital symptom experienced in the previous 4 weeks. The second part of the questionnaire asked about impact on the patient's sex life over the previous 4 weeks with an identical scoring and the third part of the questionnaire asked about a range of urinary symptoms, where the patient indicated any/all that applied.

Patient demographic information (e.g. age and smoking behaviour), clinical details (e.g. circumcision, urinary meatus type), genital symptoms and questionnaire answers were collated and analysed. Meatus type was assessed according to the criteria defined by Vieiralves et al.,^7^ into ‘normal’ or ‘abnormal’. Abnormal meatal variants were either dilated (including hypospadias and pouting) or pin‐pointed/narrow (due to scarring). Unadjusted logistic regression was used to compared meatus type and diagnostic outcome. Analyses were performed using R (Version 4.1.0) and RStudio (Version 1.1.463). A *p*‐value <0.05 was considered statistically significant.

## 3 RESULTS

Sixty four patients completed the questionnaire (surveyed patients), and 50 patients were subsequently diagnosed with MGLS. The diagnosis of the 14 non‐MGLS patients was vitiligo (2), sebaceous prominence (2), dysaesthesia (2), eczema (2), scrotal calcinosis (1), psoriasis (1), penile melanosis (1), lichen planus (1), penile intraepithelial neoplasia (1) and penile melanosis (1).

Median age at diagnosis of surveyed patients was 49 years (Interquartile range (IQR) 32–62) for MGLS patients, and 34 (IQR: 29–57) for patients diagnosed with other conditions (non‐MGLS) (Table 1). MGLS patients were more commonly uncircumcised (73% (*n* = 36) vs. 21% (*n* = 3)) and had a dilated urinary meatus (26 (52%) vs. 4 (29%)). A pseudo‐foreskin was seen in four MGLS patients (8%).

**TABLE 1:** Characteristics of patients with genital symptoms who completed the questionnaire, which includes 50 patients diagnosed with MGLS and 14 diagnosed with other conditions.

Characteristic	MGLS	Other conditions^a^
	*n* = 50	*n* = 14
Age		
Mean (SD)	47 (17)	41 (20)
Median (IQR)	49 (32–62)	34 (29–57)
Range	18–75	18–81
Total symptoms		
Mean (SD)	6 (2)	5 (3)
Median (IQR)	7 (5–8)	4 (2–6)
Range	1–11	0–11
Duration of symptoms		
Mean (SD)	33 (35)	49 (65)
Median (IQR)	24 (12–36)	24 (20–45)
Range	2–180	2–250
Dilated meatus		
Abnormal	42 (84%)	4 (29%)
Normal	8 (16%)	9 (64%)
Unknown	0 (0%)	1 (7.1%)
Circumcised		
No	36 (72%)	3 (21%)
Pseudoforeskin	4 (8.0%)	0 (0%)
Yes	10 (20%)	11 (79%)
Smoking status		
No	43 (86%)	12 (86%)
Yes	5 (10%)	1 (7.1%)
Past	2 (4.0%)	1 (7.1%)
Genital condition affecting sex life		
Yes	40 (80%)	10 (71%)
No	8 (16%)	4 (29%)
Unknown/no reply	2 (4.0%)	0 (0%)
Dyspareunia		
No problems	9 (18%)	3 (21%)
No sex	19 (38%)	6 (43%)
Reduced sex	20 (40%)	5 (36%)
Soreness with sex	2 (4.0%)	0 (0%)

^a^ Patients with other conditions (*n* = 14) were diagnosed with eczema, vitiligo, sebaceous prominence, penile melanosis, scrotal calcinosis, dysaesthesia, lichen planus, or PeIN.

The median number of total symptoms reported was 7 (IQR: 5–8) and 4 (IQR: 2–6) for MGLS and non‐MGLS patients respectively. The duration of symptoms was similar between MGLS and non‐MGLS patients, with a median duration of 24 months before clinical assessment for both (MGLS IQR: 12–36, non‐MGLS IQR: 20–45).

The association between meatus type and diagnostic outcome showed the odds of a MGLS diagnosis for patient with an abnormal meatus is 11.81 times (95% CI: 3.10–53.34, *p* < 0.001) that compared to a normal meatus.

**FIGURE 1:**
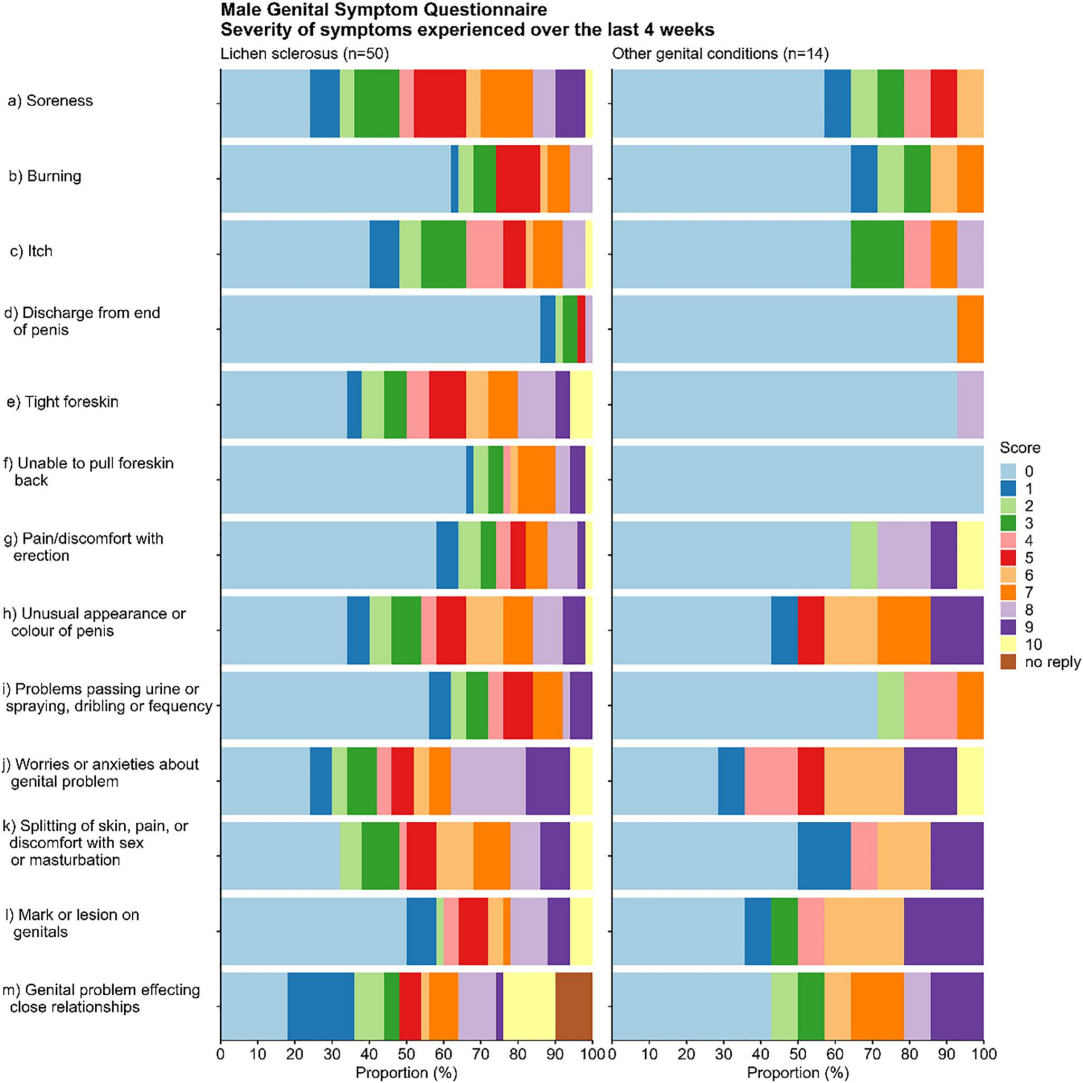
Severity of symptoms experienced by patients over the last 4 weeks rated from 0 (no effect) to 10 (severe effect), shown for MGLS and non‐MGLS patients. Each question is presented with the proportion (%) of score patient gave the symptom. MGLS, male genital lichen sclerosus.

Soreness (*n* = 38), anxiety (*n* = 38), and splitting (*n* = 34) were the most recorded symptoms amongst MGLS patients, while anxiety (*n* = 10), mark/lesion on genitals (*n* = 9), and usual appearance or colour of penis (*n* = 8) were most common amongst non‐MGLS patients (Figure 1). Of the 68.8% of MGLS patients with soreness, 29.7% (*n* = 17) score the severity as a 7 or higher. Worries or anxieties about the genital problem received the highest severity rating; 19 patients (38%) scored the severity as an 8 or higher. 41 (76.6%) patients with MGLS and 8 (57.1%) patients with non‐MGLS reported that genital skin problems had affected close relationships over the last 4 weeks.

Individual questions from the questionnaire were compared for all pairs of questions using a correlogram (Figure S1). Multiple questions showed positive correlations, for example, where scoring in tight foreskin (question e) increases so does scoring in unable to pull foreskin back (question f) (Figure S2). Soreness (question a) was correlated with burning (question b), splitting (question k), and unable to retract foreskin (question k). Painful erection (question g) was correlated with soreness (question a), splitting (question k), itching (question c), and tight foreskin (question e).

Twenty six (52%) MGLS and 3 (21%) non‐MGLS patients reported difficulties with passing urine in the 4 weeks before diagnosis (Table 2). Most common reasons among MGLS patients were small amount of dribbling immediately after passing urine (*n* = 16), passing urine more than once in the night (*n* = 12), and spraying of urine (*n* = 10). Due to low numbers there was no statistical association regarding urine dribbling and meatus type (normal or abnormal). From logistical regression, all patients (MGLS and non‐MGLS): 2.80 (0.76–13.60), *p*‐value 0.1499 and for MGLS only: 1.91 (0.38–14.30), *p*‐value 0.465.

**TABLE 2:** Urinary symptoms in the 4 weeks prior to diagnosis.

	MGLS	non‐MGLS
	*n* = 50	*n* = 14
Urinary symptoms		
None	18 (36%)	11 (78%)
Less powerful stream than before onset of genital problem	2 (4%)	1 (7%)
Spraying of urine	10 (20%)	0
Dropping of urine in underwear after passing urine	13 (26%)	0
Small amount of dribbling immediately after passing urine	16 (32%)	2 (14%)
Dribbling half an hour or longer after passing urine	0	0
Passing urine more often than before onset of genital problem	4 (8%)	0
Passing urine more than once in the night	12 (24%)	1 (7%)
Urinating problems affecting relationships	0	0
Urinating problems affecting leaving the house	1 (2%)	0
No reply	6 (12%)	0

*Note*: Patients were asked to choose all those symptoms that applied.

## 4 DISCUSSION

The symptoms associated with a MGLS diagnosis reflect the pathology of the disease. The condition causes active inflammation (giving symptoms such as pain, soreness and dyspareunia) and scarring (giving symptoms such as pain, splitting of the frenulum and foreskin, difficulty retracting the foreskin). In addition, men may get urinary symptoms such as dribbling which may be related to the anatomy of the urinary meatus.^8^ Common symptoms associated with MGLS presented in this study are largely in line with previous studies,^1^, ^9^, ^10^ however, describing the frequency and severity of reported symptoms is unique to this study.

Different studies on MGLS have described different symptoms. ‘Male dyspareunia, itch and rash’ were described as the ‘predominant symptoms’ by Kravvas (2018).^9^ In contrast, Riddell et al.^11^ found ‘tight foreskin’ and ‘difficulty passing urine’ as the most common symptoms in their MGLS patients. A retrospective questionnaire study from 2014^10^ found that MGLS patients reported only five symptoms: itch, discomfort, tenderness, pain, cosmetic disturbance, with the most common symptom being tenderness. Another frequently reported symptom is sexual dysfunction with dyspareunia.^1^, ^3^ Understanding the range of symptoms in patients is essential to provide correct clinical care.

Kantere et al.^10^ found that patients often experienced more than one symptom, (although no data was offered on how many different symptoms), and a reduction in patients' sex life. These findings agree with results presented here; MGLS patients reported a median of seven different symptoms. These results provide further support for the importance of evaluating the range of presenting symptoms.

The British Association of Dermatologists guidelines for lichen sclerosus recommends undertaking ‘a full history for all people with lichen sclerosus, including dyspareunia and psychosexual symptoms’.^12^ However, the exact symptoms to elicit are not mentioned. We propose a list of the most common symptoms are added to this guidance.

To date, there has been limited research on symptom severity scoring in MGLS. One study used the Dermatology Life Quality Index (DLQI) and PGI‐1 (Patient Global Assessment of Improvement) tools to assess patients' quality of life in order to assess the impact of a single therapy for MGLS.^13^ The DLQI, a recognized quality of life questionnaire, is without disease‐specific focus and does not reveal specific symptoms. The questionnaire used here was developed by the lead author who is an expert in the field with extensive experience of treating patients with genital symptoms.

The questionnaire allowed patients to self‐reported symptoms using a numeric rating scale from 0 (no effect) to 10 (severity effected). Self‐reported numeric rating scales are commonly used in a range of medical settings such as in pain assessment and management.^14^ Numeric scales have the advantage of being quick to administer and score, and usually score symptoms from 0 to 10. They have been used in dermatology for assessment of single item itch severity in atopic dermatitis.^15^ The questionnaire is intended to be used for assessment at the initial appointment and continued monitoring during follow‐up. The analysis and visualization of the severity of symptoms reported here provides a unique insight into this cohort. Although this study is based on a smaller number of patients compared to some prior studies, this work offers valuable insights through the availability of questionnaire data on all patients. This study was limited to patients seen in a specialist male genital clinic, making these findings potentially less generalizable. Patients with less severe symptoms or disease could be treated by other clinical specialties and might be less likely to be seen in the specialist clinic.

A further study could assess the association between MGLS symptoms and patient specific outcomes after treatment, including subsequent development of genital skin cancer.

Many doctors are trained to ask only about the main physical symptoms of a condition (the history of the presenting complaint) rather than assess the full range of the patient's symptoms. This approach may fail to address issues that may be the patient's main concern. MGLS is a complex condition that is potentially pre‐cancerous and often requires a multi‐disciplinary approach to management. Patients may present to a range of specialties (sexual health, general practice, urology, dermatology) so a unified approach to management is needed. We believe a self‐reported questionnaire examining the patient's full range of symptoms, urological health and sexual health is the best approach.

Male genital lichen sclerosus has an impact on patient's sexual health and quality of life. ‘Worries and anxiety about genital problem’ was amongst commonly reported symptoms found here. To provide accurate care and diagnoses it is essential for clinicians to understand the physical symptoms and psychological components of this condition. This study provides an important insight into which symptoms and their severity are common amongst patients with lichen sclerosus. In addition, the questionnaire used here can provide a basis for other clinicians in their assessment of MGLS patients.

## CONFLICT OF INTEREST STATEMENT

BvB is an employee of the British Association of Dermatologists.

## AUTHOR CONTRIBUTION

**Manu Shah**: Conceptualization (equal); Data curation (equal); Supervision (equal); Writing – original draft (equal); Writing – review & editing (equal). **Birgitta van Bodegraven**: Data curation (equal); Formal analysis (equal); Writing – original draft (equal) Writing – review & editing (equal).

## ETHICS STATEMENT

Not applicable.

## Supporting information

Supplementary Material

## Data Availability

Raw data and a copy of the questionnaire can be requested through a data request with MS.
