# Supplementary material for: Male genital lichen sclerosus and associated symptoms range and severity: Results of a questionnaire study
Source: Skin Health Dis. 2023 May 12;3(5):e246. doi: 10.1002/ski2.246 (PMC10549852; doi:10.1002/ski2.246)
Supplement: Supplementary file 1 — Supplementary Material [file SKI2-3-e246-s001.docx]

**Supplementary information:**


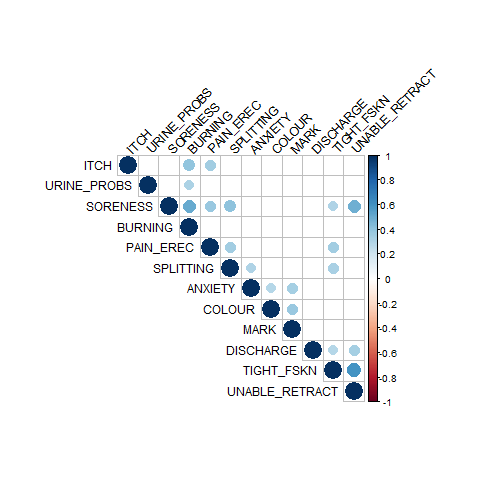


Figure S1. Exploratory comparison of relationship between questionnaire questions shown as a correlogram. All combinations are evaluated but only those with P<0.05 are shown as blue dots. The strengths of associations can be seen as the size and colour of the blue dots.

**
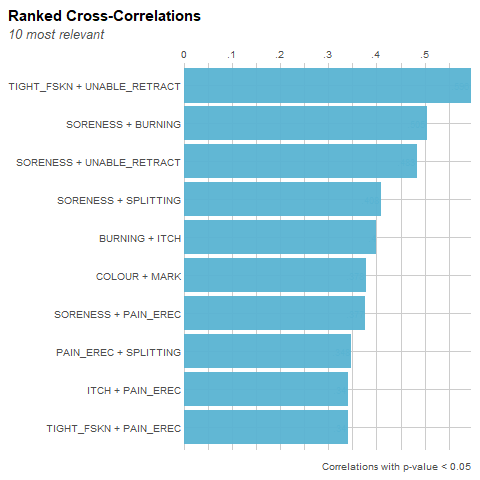
**

Figure S2. Comparison of most relevant questionnaire questions for MGLS patients through ranked cross-correlation of all questions. Figure shows the 10 most relevant combinations based on correlation and p-value.
